# Facile synthesis of gold nanocages with silver nanocubes templates dual metal effects enabled SERS imaging and catalytic reduction†

**DOI:** 10.1039/d3ra06344e

**Published:** 2023-10-27

**Authors:** Farukh Mansoor, Huangxian Ju, Madiha Saeed, Shamsa Kanwal

**Affiliations:** a Key Laboratory of Magnetic Materials and Devices & Division of Functional Materials and Nanodevices, Ningbo Institute of Materials Technology and Engineering, Chinese Academy of Sciences Ningbo 315201 P. R. China; b Department of Chemistry, Khwaja Fareed University of Engineering and Information Technology Abu Dhabi Road Rahim Yar Khan Pakistan shamsa.sham@gmail.com; c State Key Laboratory of Analytical Chemistry for Life Sciences Department of Chemistry, Nanjing University Nanjing 210023 China; d Interdisciplinary Research Centre in Biomedical Materials, COMSATS University, Islamabad, Lahore Campus Lahore Pakistan

## Abstract

Silver (Ag) nanomaterials featuring a cubic shape particularly represent supreme class of advance nanomaterials. This work explored a new precursor and its effect on morphological features of silver (Ag) nanocubes (NCs) serving as sacrificial templates for facile synthesis of gold NCs. The AgNCs were initially prepared utilizing sodium thiosulphate (Na_2_S_2_O_3_) as relatively stable S^2−^ producing species along with a soft etchant source KCl. The effects of different potassium halides were evaluated to grasp control over seed mediated growth of Ag nanocubes. Taking the advantages of dual metallic properties, Ag@4MBA@AuNCs nanostructure was synthesized using 4-mercaptobenzoic acid (4MBA) as a Raman reporter molecule. This nanostructure showed 10^10^-times enhancement in surface enhanced Raman scattering (SERS) signal, leading to a highly sensitive imaging probe for the detection of even three breast cancer cells (MCF-7 cells) *in vitro*. Subsequently, the oxidative nanopeeling well accompanied by incorporation of Au/Ag alloy nanoparticles on AuNCs corona assembly was achieved, which facilitated the catalytic reduction of toxic nitrophenol to eco-friendly aminophenol. Such sophisticated and engineered nanoassemblies possess broad applications in bioanalysis.

## Abstract 2

Silver (Ag) nanomaterials featuring a cubic shape particularly represent supreme class of advance nanomaterials.

## Graphical abstract

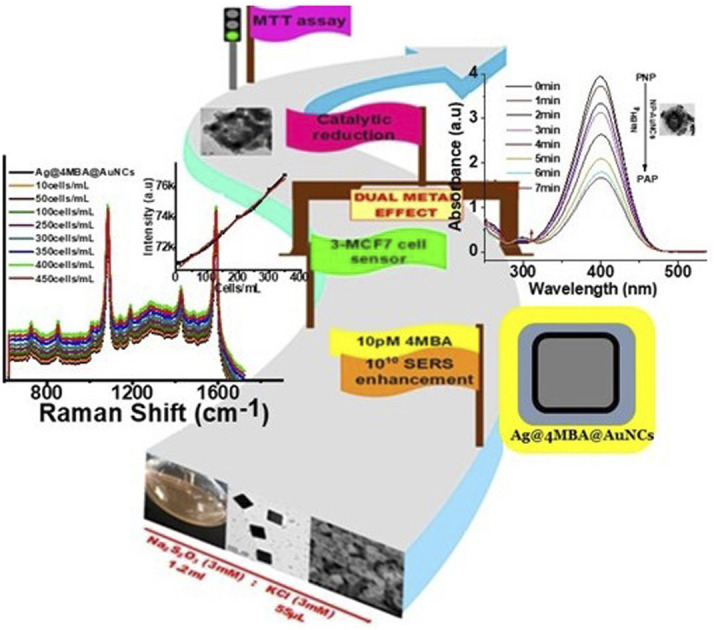

## Introduction

Among all nano-architectures explored so far intense research has been directed towards the development and refinement of protocols used for the synthesis of Ag nanocrystals with a broad range of sizes and shapes.^1–10^ Controlling the shape of colloidal metal nanocrystals is exclusively important to realize their diverse applications in catalysis, photonics, electronics, and biomedicine. Ag nanocubes have garnered particular attention due to their superb performance in applications involving localized surface plasmon resonance (LSPR)^1–4^ and surface-enhanced Raman scattering (SERS). In comparison to other solid nanostructures, the hollow ones are propitious to applications as they enable robust diffusion of chemicals from and to confined reactive sites, high surface-to-volume ratio and large pore volume substantial to increased catalytic activity, energy storage/conversion and release of payload. In this regard tuneable edge lengths and sharp corners/edges of Ag nanocubes serve as sacrificial templates to fabricate AuNCs which in turn become outstanding dual metal substrates for optical sensing and SERS imaging^5–8^ with tuneable LSPR for diverse applications in photothermal therapy,^9,10^ photodynamic therapy,^9^ drug delivery^11^ and gene therapy.^10^

Polyol-mediated synthesis of Ag nanocubes is positioned among pioneer mechanisms facilitating the production of twin seeds well controlled through polyol reduction. Subsequently, single crystal seeds are attained through and grown into nanocubes in the presence of oxidative etchant and hydrophilic solvents.^12^ Nevertheless, the polyol synthesis is sensitive to both impurities like Fe^2+^/Fe^3+^ ions,^3^ water, oxygen, and Cl^−^ ions^13^ and the lengthy protocols extending up to 2 days in the presence of soft etchant like NaCl, which make the robust synthesis greatly challenging.^14^ Sulphide assistance, practically the highly reactive S^2−^ species (NaSH or Na_2_S),^2^ drastically initiates the single crystal growth of Ag nanocubes in the presence of strong etchant like HCl.^3^ It can reduce the synthesis time to less than 10 minutes.^3^

However, halides like KBr,^15^ KI,^1^ NaBr^16^ and NaCl^17^ have also been typically used to attain morphological control over Ag nanocubes. By simply substituting NaCl with NaBr in polyol-mediated synthesis, right bipyramids with purity yield of 80% have been produced instead of Ag nanocubes.^18^ However, exploration of KCl and closely related salts like KBr and KI in combination with mild reducing agent still remains unapproached. Herein we report an efficient protocol utilizing sodium thiosulphate (Na_2_S_2_O_3_) and KCl as reducing agent and etchant respectively, gradually producing monodispersed single crystal Ag nanocubes with sharp edges and corners. Relatively stable S^2−^ producing species (Na_2_S_2_O_3_) and soft etchant like KCl facilitated the monitoring of argon free robust synthesis process at regular intervals, even for minute changes in the appearance of reaction mixture. The UV-vis and TEM were used for back-to-back evaluation of size growth and morphological changes, and XRD confirmed excellent yield of Ag nanocubes *i.e.* up to 85%. On the other hand, sensing and imaging technologies based on supersensitive SERS have been developed for monitoring of even single molecule based on the properties of metallic surface.^19^ Metallic substrates such as Au and AgNPs,^19^ nanorods,^20^ nanostars^21^ and nanocubes^22^ have been used as successful SERS substrates manifesting potential applications in photoacoustic imaging, MIR, CT and chemotherapy.^23–26^ Ag is superior to Au in terms of the SERS performance by virtue of the strong interparticle near field coupling effect and the sharp plasmonic peak.^21,27^ Ag nanocubes with sharp corners and edges can enhance the Raman scattering cross section by 10^7^–10^8^ fold in terms of enhancement factor (EF).^28^ Moreover, it is still highly desirable for ultrasensitive assays to design SERS substrate that combines facile and versatile synthesis with extraordinary SERS enhancement. Meanwhile, various reports have already established the potent catalytic behaviour of AuNCs owing to their lower activation energy, continuously conductive thin walls, hollow structures and large surface area-to-volume ratio.^29^ Multiple holes at the corners of the NCs serve as catalytic sites.

In this work, taking advantage of dual metal effect of purposely built nanoprobes (Ag@4MBA@AuNCs) the SERS enhancement factor up to 10^10^ folds has been achieved. Resultantly, ultrasensitive detection of Raman reporter molecule 4MBA was also attained up to 10 pM levels. Significantly, the intensified SERS signals led highly sensitive SERS-active *in vitro* imaging probes (Ag@4MBA@AuNCs) capable to monitor at least 3 MCF-7 cells. However formulation of nanopeeled assembly consisting of nanocages decorated with bimetallic nanoparticles may offer better results for catalytic reduction well monitored through LSPR as well as SERS. The effective catalytic performance of NP-AuNCs was assessed by reduction of toxic nitrophenols to aminophenols well justified by UV and Raman spectra thus opening avenues for environmental monitoring.

## Experimental

Detailed information about the materials, instruments used for characterization and synthesis routes of various assemblies has been detailed in ESI.† The detailed synthetic procedure to fabricate Ag nanocubes from facile precursor combination with enough reproduction details has been described below.

### Synthesis of Ag nanocubes

Briefly, in a 500 mL three-neck round bottom flask 100 mL of ethylene glycol (EG) was heated at 150 °C with vigorous stirring (1100 rpm) on an oil bath for 50 min. Then 1.2 mL freshly prepared solution of 3 mM Na_2_S_2_O_3_ and 55 μL of 3 mM KX solution (KCl/KBr/KI) were injected into hot EG in separate experiments. After 2–3 min, 25 mL of PVP solution (20 mg mL^−1^) was injected into the reaction mixture. At this stage, the temperature was adjusted to 150 °C and CF_3_COOAg (0.622 g in 8 mL EG) was drop-wise added to this reaction mixture. Colour of the reaction mixture changed with addition of white drops turning a bit yellow, which later turned orange, thick grey, dirty green, and finally to reddish brown. The solution was then cooled to room temperature, and precipitates of Ag nanocubes were separated, washed thrice with acetone and deionized water once, and centrifuged at 12 000 rpm for 15 min to remove unreacted precursors. Ag nanocubes of different sizes (ranging from 50–150 nm) were synthesized by following similar procedure with different reaction time intervals up to 40–45 min.

### Developing 4MBA@nanoassemblies for SERS enhancement

Two sets of 4MBA coated nano-assemblies *i.e.* AuNCs@4MBA and Ag@4MBA@AuNCs were synthesized as per previous reports^19^ with some sequence modifications as follows: briefly, AuNCs@4MBA (set A) were prepared by one step coupling in which 50 μL of 0.05 mM 4MBA was added dropwise while stirring into the as synthesized AuNCs for another 10 min. Another titration with HAuCl_4_ as mentioned above was conducted to add another layer of gold over 4MBA coated AuNCs, thus producing 4MBA@AuNCs. Likewise for comparison, Ag@4MBA@AuNCs (set B) were synthesized when 1 mL of the Ag nanocubes of 120 nm edge length were added into 8 mL of deionized water under stirring, and 30 μL of 0.05 mM 4MBA was added dropwise to form 4MBA coated Ag nanocubes. After 15 min these Ag@4MBA were titrated against HAuCl_4_. Finally, two sets of nano-assemblies containing AuNCs@4MBA and Ag@4MBA@AuNCs were separately collected by centrifugation at 4000 rpm/5 min and washed with deionized water three times before re-dispersion.

### SERS based tumour imaging (*in vitro*)

Raman spectra of pure AuNCs and cells incubated with 4MBA@AuNCs as well as Ag@4MBA@AuNCs were recorded by a Raman spectrometer. For this purpose, both 4MBA@AuNCs and Ag@4MBA@AuNCs (40 μg mL^−1^ each) were dispersed in PBS separately, and their Raman spectra were measured. For Raman spectral investigation of SERS imaging based on set A and set B, MCF-7 cells in logarithmic growth were cultured in 35 mm culture dishes for 24 h and then incubated with 4MBA@AuNCs and Ag@4MBA@AuNCs in PBS formulation for another 4 h. Finally, the cells were washed with PBS thrice, mixed with paraformaldehyde, and the Raman spectra were recorded at 785 nm with the laser power of 14 mW (integration time = 10 s).

The MCF-7 cells were grown in logarithmic way, cultured in 35 mm culture dishes for 24 h. After 24 h, for a series of SERS imaging protocols, MCF-cells were incubated with pure 4MBA (1 μM in PBS), pure AuNCs (40 μg mL^−1^ in PBS) and pure Ag nanocubes, as well as with 40 μg mL^−1^ of each sets *i.e.* 4MBA@AuNCs and Ag@4MBA@AuNCs for another 4 h. After incubation, the cells were washed with PBS three times and mixed with 2% paraformaldehyde solution before measurements on Raman spectrophotometer @ 785 nm. The concentrations of MCF-7 cells were 50 cells per mL for pure AuNCs, and 10–450 cells per mL for both 4MBA@AuNCs as well as Ag@4MBA@AuNCs.

### Cell culture and cytotoxicity assay

Methyl thiazolyl tetrazolium (MTT) assay was used to evaluate the cytotoxicity of 4MBA@nanoassemblies for SERS imaging. In DMEM culture medium, MCF-7 cells were first cultured with 10% FBS using 100 units per mL penicillin and 100 mg mL^−1^ streptomycin in 5% CO_2_ at 37 °C. MCF-7 cells were seeded at a density of 1 × 10^4^ cells per well in a 96-well culture plate and incubated for 24 h at 37 °C with 5% CO_2_. Then, various concentrations of 4MBA@AuNCs and Ag@4MBA@AuNCs (0, 25–500 μM) were added to each well. After 24 h of incubation, 10 mL of MTT solution (5 mg mL^−1^) was added and incubated for 4 h. Subsequently, the media were removed, followed by the addition of 100 mL of dimethyl sulfoxide (DMSO) to dissolve the formazan crystals. Finally, the absorption of each well was measured using a microplate reader (iMark 168-1130, Bio-rad, USA) and the cell viability was calculated.

### Nanopeeling and photocatalytic reduction of nitrophenols

Firstly, 5–20 mg dopamine was dissolved into 10 mL of Tris buffer (5 mM, pH 8.5). Then, 10 mL of AuNCs were quickly injected into dopamine solution. The sonication time was adjusted about 10 min to obtain polydopamine coated AuNCs (PD@AuNCs). PD@AuNCs were purified by centrifugation at 7000 rpm for 10 min (twice) and were redispersed in 10 mL distilled water, respectively. Half titrated AuNCs with the size 120 nm in diameter were reacted in 10 mM Tris buffer with different concentrations of dopamine for 4 h at pH 8.5 to obtain 3–25 nm thick PD layer over AuNCs (PD@AuNCs). In subsequent steps, titration of PD@AuNCs with 1 mM each of HAuCl_4_ 50–250 μL and triethyl amine (TEA) was performed to synthesize NP-AuNCs. Later, in 500 μL PNP (1 μM), 0.5 mL freshly prepared solution of NaBH_4_ (1 mM) and 30 μL NP-AuNCs were added, and UV spectra were measured after every minute till next 15 min. The reduction of nitrophenols was also determined using NP-AuNCs as per afore-mentioned procedure by using Raman spectroscopy to assure the practical impacts.

## Results and discussion

Traditionally, Ag nanocubes utilize fine proportion of EG, PVP and sulphide (S^2−^) or bisulfide ions (SH^−^)^17^ coming from highly reactive sources such as Na_2_S^3^ and NaHS,^2^ respectively. This protocol offers argon free robust synthesis comparative to several reports where trace amounts of O_2_ could greatly influence the process and yield of the reaction. O_2_ already existing in the reaction solution and Cl^−^ ions from KCl were validated as a typical example for generating single-crystal seeds and thus Ag nanocubes.^13^ Herein we used Na_2_S_2_O_3_ under acidic conditions that dissociates into free S atoms to react with AgCl matrix forming Ag_2_S initially. As produced Ag_2_S clusters serve as primary nucleation sites to form single-crystal Ag-seeds.^30^ Once the concentration of Ag-seeds attains the supersaturation value, they start to nucleate and grow into single-crystal cube.^14^ As per reports during synthesis without aid of X^−^ = Cl^−^, Br^−^, I^−^ and in the presence of oxygen, Na_2_S_2_O_3_ acts as an etchant on AgNPs, converting their morphology from triangular nanoprisms to hexagonal nanoplates.^31^S_2_O_3_^2−^ → S_(s)_ + SO_3_^2−^_(g)_ + SO_4_^2−^_(g)_2AgCl + S → Ag_2_S_(s)_

In our approach Na_2_S_2_O_3_ known for stability^32^ was combined with Cl^−^ ions of KCl to envisage their effect on morphological features of Ag nanocubes. Success of this synthesis relies on a tight control over the nucleation process through the addition of appropriate amount of KCl as the Cl^−^ source and Na_2_S_2_O_3_ as reducing agent. Cl^−^ ions play two critical roles in the nucleation and growth of Ag nanocubes with a single-crystal structure. First, the Cl^−^ ions react with Ag^+^ ions to generate nanometer-sized AgCl octahedra in the initial stage of synthesis^33^ avoiding hexagonal nanoplates. Production of white coloured AgCl upon addition of Na_2_S_2_O_3_, PVP and KCl into the reaction mixture corresponds well with already published reports during formation of Ag nanocubes.^17^KCl + CF_3_COOAg → CF_3_COOK + AgCl_(white ppt)_

**Fig. 1:**
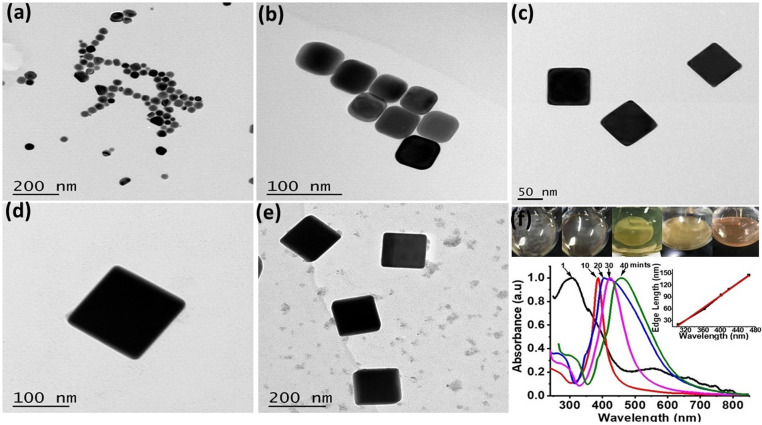
TEM images of Ag nanocubes at various reaction times from (a)–(e): (a) 1, (b) 10, (c) 20, (d) 30 and (e) 40 min; (f) reaction mixture images at different time intervals inset represents linear relation of wavelength with edge length at different time intervals.

Within 1–2 min, white precipitates instantly turn to blackish grey intimating Ag_2_S formation before abrupt turn to yellow to orange indicating the production of silver nanoparticles (AgNPs). These AgNPs served as Ag-seeds in the reaction mixture, which on prolonged heating and stirring for 40–45 min produced Ag nanocubes (Fig. 1a–e). Basic reaction involves the reduction of Ag from CF_3_COOAg at 150 °C in EG.^4^ Upon addition of Na_2_S_2_O_3_, PVP and KCl into the reaction mixture, various reaction stages are recognizable through colour changes as shown in Fig. 1f. Immediately after addition of CF_3_COOAg, the solution changed white in colour, followed by the appearance of a slight yellow colour after 2–3 min and it remains yellowish before turning to deep orange. The molar ratio (PVP : CF_3_COOAg, 2 : 1) was maintained to obtain Ag nanocubes in high yields. Subsequently, Ag nanocubes started appearing in the solution as indicated by a shiny metallic layer formation on the walls of reaction flask with a brown coloured reaction mixture. TEM images and corresponding LSPR peaks of the Ag nanocubes formed at 1, 10, 20, 30 and 40 min (Fig. 1a–e) clearly indicate that Ag nanoseeds of ∼20 nm size were produced instantly having *λ*_max_ at 300 nm. Gradual increase in edge length (45, 85, 110 and 145 nm) was observed. Actually, reaction time gradually contributes to edge length increase of Ag nanocubes and observed moderate growth rate equally facilitates a quick and on time UV/Vis spectral evaluation of small aliquots sampled from the reaction solution.

For in depth understanding of the synthetic progress of Ag nanocubes, LSPR peak positions at different time intervals were monitored and corresponding LSPR peak positions (Fig. 1f) were exhibited at 303, 373, 410, 438 and 465 nm respectively. Major change in the LSPR peak position during the initial 10 min was recorded and single peak positioned at 320 nm red shifted to 390 nm was observed. This is attributed to the transformation of Ag nanoseeds to Ag nanocubes of the size ∼45 nm well correlated with previous findings.^17,34^ Noticeably, a linear relationship between the LSPR peaks position and edge lengths of Ag nanocubes was observed (inset in Fig. 1f) with linear regression value of *R*^2^ = 0.99.

This protocol offers argon free robust synthesis comparative to several reports where trace amounts of O_2_ could greatly influence the process and yield of the reaction. O_2_ already existing in the reaction solution and Cl^−^ ions from KCl were validated as a typical example for generating single-crystal seeds and thus Ag nanocubes.^13^

To reveal the role of best halide ion selective etching through HCl remained dominant mechanism for producing nanocubes along with additional elements that contribute to its success.^14,17^ Whereas, role of various halide ions such as (Cl^−^, Br^−^, I^−^) for morphological control of Ag nanocubes^16,33,34^ guided to explore effect of both protonic and anionic behaviours of different potassium halides (KCl, KBr and KI). Results reveal minute changes in KCl concentrations greatly effect the morphological control. 55 μL of 3 mM KCl (Fig. S1†) precisely controlled cubic morphology as compared to KBr and KI counterparts (Fig. S2†) in the order of KCl > KBr > KI. Importantly, EG and PVP along with KBr were previously used to synthesize silver nanobars.^35^ In this case Fig. S2† reveals formation of silver nanorods alongside few nanocubes of silver well confirming the etching and reduction behaviour of using S_2_O_3_^2−^ in combination with halide ions. Undoubtedly, the K^+^ ions in KCl exhibit the superior role as compared to that of H^+^ ions in HCl, which reportedly slow down the reduction process generating more single crystal seeds.^36^ Owing greater oxidation potential than H^+^ ions,^37^ we believe that K^+^ ions may act as strong reducing agents, meanwhile Cl^−^ ions act as relatively soft etchant as compared to HCl^14^ facilitating the synthesis of single-crystals Ag nanocubes at elevated temperature.^14^ It has also been reported that Cl^−^ ions absorb more strongly on Ag (100), having strong etching property than Br^−^ and I^−^, causing shape control of silver nanoparticles ranging from truncated octahedra to cuboctahedra, truncated cubes, and eventually cubes; correspondingly, *ab initio* thermodynamic calculations support the formation of cubes from Wulff shape.^38^

PVP acts as a capping agent that selectively binds to the (100) facets of Ag-crystal and favors the formation of Ag nanocubes.^39^ This combination is so far unexplored to develop Ag nanocubes, therefore, to obtain high quality Ag nanocubes with improved yield, optimization was done by precise adjustment of concentrations of both Na_2_S_2_O_3_ and halogen salts as mentioned in Tables S1, S2, Fig. S1 and S2.† Principally, the concentrations of Na_2_S_2_O_3_ and KCl were optimized to achieve nanocubes with perfect edges and truncated corners. Results manifest that if KCl concentration was raised up to 17 μM, the Ag nanocubes show severe truncations at the corners and edges, beyond which other morphologies such as bipyramids, nanorods and irregular nanomaterials were observed due to enhanced oxidative etching (Fig. S1 and Table S1†). To ensure the crystallinity of as-synthesized Ag nanocubes XRD analysis (Fig. S3†) exhibits four prominent diffraction peaks at (111), (200), (220) and (311) revealed planes of pure fcc Ag-crystals.^40^ The (111) peak intensity was relatively higher as compared to that of (200) peak, indicating a slightly truncated cubic morphology.^4^ The abundance of Ag nanocubes with facets was validated by comparing the intensity ratio of the (200) and (111) peaks of the Ag nanocubes with that of conventional value (0.35 *versus* 0.4).^41^ The strong (220) peak is ascribed to the substantial number of {110} facets on the nanocubes of Ag.^42^

**Fig. 2:**
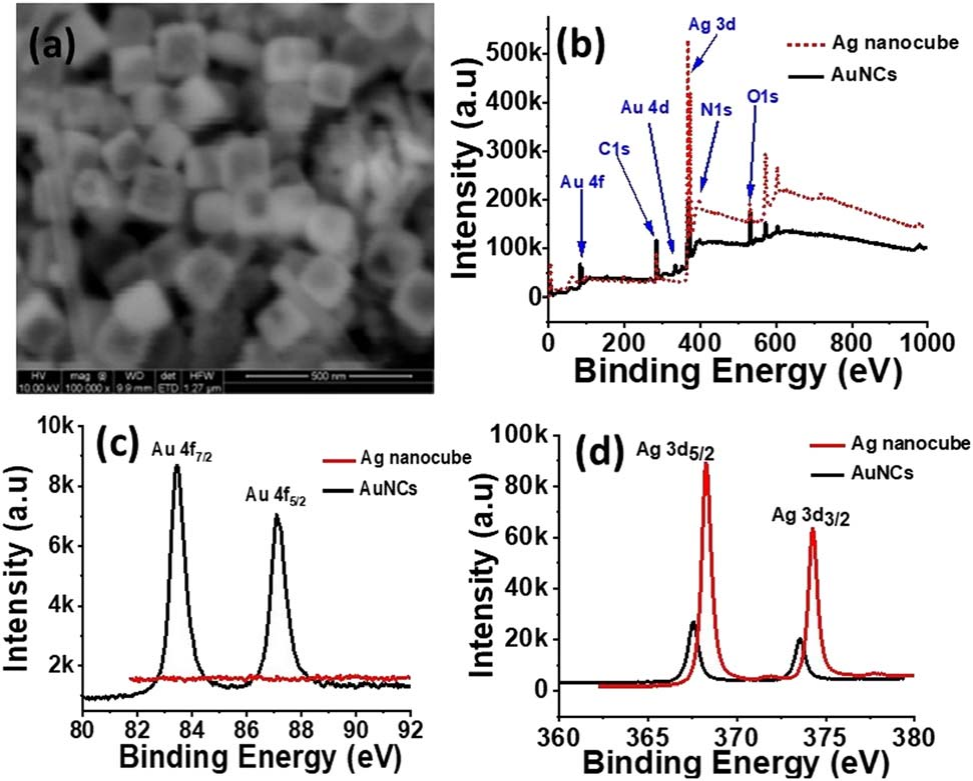
(a) SEM image of as synthesized AuNCs, (b) XPS spectra of Ag nanocubes and AuNCs, (c and d) HR-XPS spectra of Au (4f) and Ag (3d) in Ag nanocubes and AuNCs, respectively.

In next and final phase of synthesis, as synthesized Ag nanocubes were used as template to synthesize AuNCs by utilizing already established protocols^3,43^ of galvanic replacement reaction. The SEM image (Fig. 2a) represents the sharp edges and truncated corners of Ag nanocubes with edge length of 150 nm. The high-resolution (HR) X-ray photoelectron spectroscopy (XPS) spectra of Ag nanocubes and AuNCs (Fig. 2b–d) shows that Ag 3d peak, is the most prominent in case of Ag nanocubes as compared to that in AuNCs. Evidently, Au 4d and 4f peaks are present in XPS spectra of AuNCs that are clearly absent in Ag nanocubes. Moreover, the HR-XPS of Au 4f does not show any trace of Au in Ag nanocubes however for AuNCs, the two peaks of Au 4f_7/2_ and Au 4f_5/2_ have been observed exhibiting lower binding energy values (83.44 and 87.09 eV) than standard binding energy of Au (84.0 and 87.7 eV).^44^ HR-XPS spectra of Ag 3d_5/2_ and Ag 3d_3/2_ in case of AuNCs has been slightly shifted towards lower energy *i.e.* 367.5 and 373.5 eV as compared to that of Ag nanocubes which shows Ag 3d_5/2_ and Ag 3d_3/2_ peaks at 368.2 and 374.2 eV in line with the standard binding energy of Ag metal.^45^ This shift is likely due to the electron transfer from Au → Ag during the coating process of Au on Ag cubes.^46^

Based on the SERS technique along with advantage of noble metallic properties of Ag and Au in this case, we magnified spectroscopic signals of single reporter molecule of 4-mercaptobenzoic acid (4MBA). Reportedly, Au is resistant to oxidation and shows excellent biocompatibility, but its performance in plasmonic applications mainly SERS lags behind Ag by an order of magnitude. So as a rule of fact one can endorse materials both the chemical stability coming from Au forming conformal thin shell and improved SERS activity endowed from Ag in AuNCs.^1,7^ However, due to possible loss of Ag during galvanic replacement during AuNCs formation SERS activity is anticipated to deteriorate. Therefore, we opt for a strategy to maintain inner Ag layer by adhering 4MBA onto the surface of Ag nanocubes thus overruling the complete galvanic replacement ultimately obtaining 4MBA layer in between Ag and Au in hollow AuNCs with strong SERS activity.

**Fig. 3:**
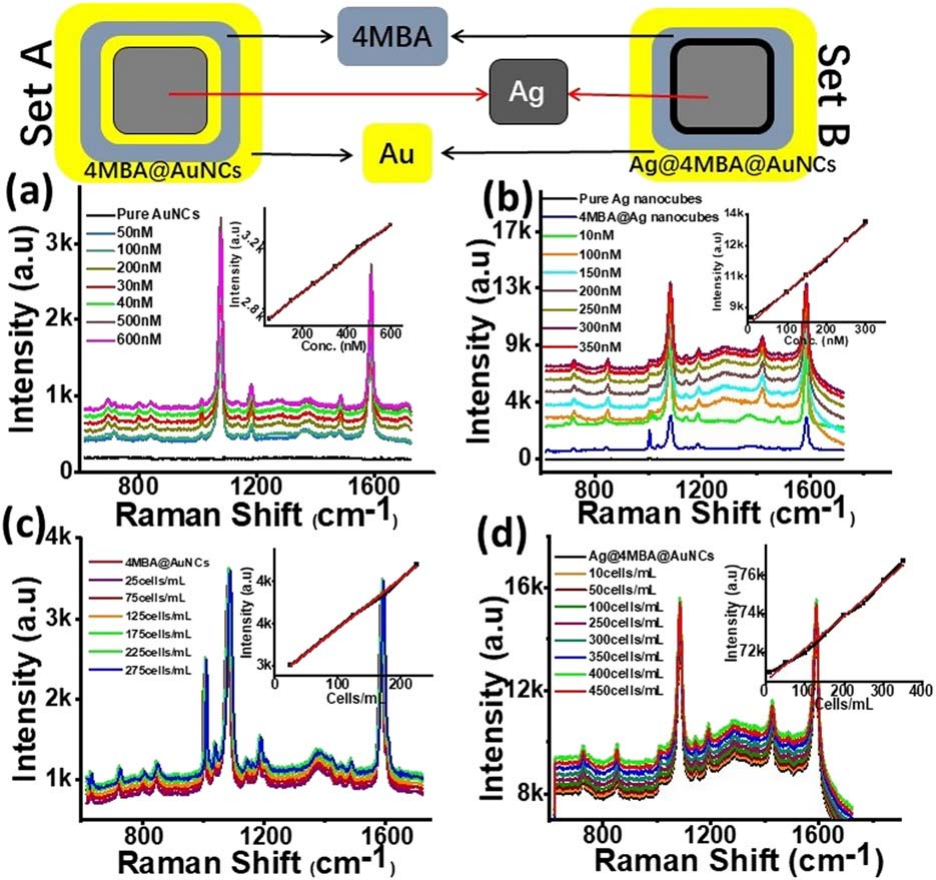
Schematic representation of 4MBA@AuNCs (set A) and Ag@4MBA@AuNCs (set B) and corresponding Raman spectra of (a) pure AuNCs, different concentration of 4MBA (50 nM to 60 μM) in set A; (b) pure Ag nanocubes, different concentration of 4MBA (10 nM to 40 μM) in set B; (c) pure set A (50 μM, 4MBA) and after incubation with 25–275 cells per mL; (d) pure set B (30 μM, 4MBA) and after incubation with 10–450 cells per mL. Conditions: 120 nm Ag nanocubes (1 mL), AuNCs (1 mL), SERS spectra were taken after incubating both sets with MCF-7 cells for 4 h, laser power = 14 mW, integration time = 10 s, insets represent linear regression equations having *R*^2^ = 0.99 in each set.

For this purpose, the SERS enhancement behaviour of two slightly different nanoassemblies *i.e.* 4MBA@AuNCs (set A) and Ag@4MBA@AuNCs (set B) were monitored for SERS enhancement evaluation (Fig. 3). Appearance of Raman bands (635, 693, 717, 800, 842, 934, 1012, 1077, 1138, 1179, 1273, 1373, 1420, 1482, 1588 and 1656 cm^−1^) in 4MBA coated nanoassemblies (sets A and B, Fig. 3a and b) were clearly distinguished from pure or 4MBA free Ag nanocubes and AuNCs, thus confirming the loading of Ag nanocubes and AuNCs with the Raman reporter molecule. It is already established fact that long range SERS effect does not require the direct contact in between adsorbate and substrate but, requires it to be within few nano-meter range of metallic substrate interface.^47^ Therefore, we believe that part of the 4MBA molecules showing SERS effect remain in close contact with the Ag/Au surface using the COO^−^ group in tilted orientation. Whereas the other parts are stacked (π–π stacking interaction) on top of the first layer of 4MBA molecules. The relative SERS under neutral conditions at 846 and 719 cm^−1^ were observed due to the metallic surface bearing 4MBA and were assigned to *δ*(COO^−^) bending vibration and *γ*(CCC), out-of-plane ring vibration, respectively.^6,48^

On the basis of bands appearing at 1077 cm^−1^ and at 1588 cm^−1^ due to the C–H in plane bending and C–C stretching of 4MBA, the SERS enhancement factor of set B was calculated to be 1.2 × 10^10^. The SERS enhancement in set B is 12 times higher than that of set A (1.0 × 10^9^). This enhancement attributes to the simultaneous exposure of Raman reporter molecule with two metals of individual properties *i.e.* Ag on inner side and Au on outer surface as shown in Fig. 3 (set B). In addition to the introduction of SERS reporter molecule between interfaces and surfaces of two metals of individual properties, its concentration effect was also monitored to achieve highest SERS signals. For this purpose, varying concentrations of 4MBA were used to monitor the effect on SERS signals. The concentration of 30 μM 4MBA in set B produced best results by rapidly enhancing SERS signals up to 12 times compared to their almost double counterparts *i.e.* 4MBA@AuNCs with 50 μM 4MBA. This is possibly due to the strong SERS enhancement endowed from Ag phase. Comparatively lower SERS in case of set A is also likely due to loss of Ag content during galvanic replacement.

Using 4MBA as probe molecule, SERS effects of both sets were investigated in a wider range of 4MBA concentration and the substrates allowed the detection of 4MBA with LOD as low as 10 pM. The SERS peaks of set A (Fig. 2a) were monitored at 1077 cm^−1^ and it was observed that SERS signals intensity increased continuously up to 50 μM while varying the volume of 4MBA from 50 nM to 60 μM. Almost similar trend with halved concentration of 4MBA was observed in set B (Fig. 2b). The SERS signals intensity was observed to increase consistently up to 30 μM in the concentration range 10 nM to 35 μM. The results endorsed both developed sets (set A & set B) to be applied as highly sensitive sensor for 4MBA in the concentration range from 50 nM to 0.6 μM, 10 nM to 3.5 μM with LOD of 50 and 10 pM, respectively having linear regression coefficient of 0.99 in both cases.

Subsequently, taking advantage of greatly intensified SERS signals of 4MBA at the interface of metals of different properties, this highly sensitive protocol for 4MBA itself led our way towards exploring potential applications of both sets as highly sensitive SERS-active imaging probe for MCF-7 cells. For this purpose, *in vitro* SERS spectra of MCF-7 cells were measured by incubating set A with 25–275 cells per mL and set B with 10–450 cells per mL for 4 h. It is clearly shown (Fig. 3c and d) that SERS signal intensities increased gradually along with rise in concentrations of MCF-7 cells up to 225 cells per mL in case of set A and for set B highest SERS signals were measured with the cell count of 350 cells per mL, beyond which signals were quenched. Insets (Fig. 3c and d) shows a linear relationship between SERS intensity and the MCF-7 cell concentration in the range of 25–225 cells per mL and 10–400 cells per mL with LOD as low as 3 cells per mL when calculated for set B. This protocol demonstrates the potential application of proposed SERS sensor at an early stage for quantitative analysis (*in vitro*) of breast cancer cells.

To further investigate the utilization of 4MBA@AuNCs and Ag@4MBA@AuNCs in bio-applications cytotoxicity test was performed. In a traditional MTT assay it was observed that unlike previous study^49^ both sets of nanoassemblies do not cause significant toxicity upon incubation with MCF-7 cells even up to 500 μg mL^−1^ as shown in Fig. S4† when measured on 550 nm. Thus, Ag@4MBA@AuNCs can be safely utilized up to 500 μg mL^−1^ for *in vivo* SERS based assessments.

**Fig. 4:**
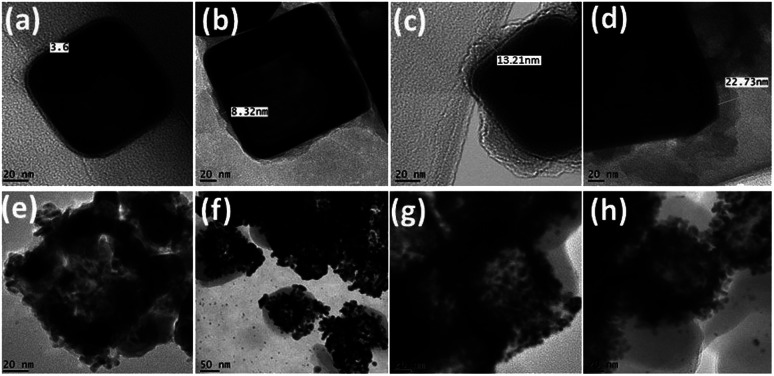
TEM images of (a–d) polydopamine (5–20 mg) corona formation around AuNCs; (e–h) Au/AgNPs corona around nano-peeled AuNCs.

To introduce multifunctionality and to enhance the catalytic activity of as-synthesized AuNCs, a rare phenomenon of nanopeeling was investigated after coating AuNCs with polydopamine (PD). For this purpose, half-titrated AuNCs of the size 120 nm were reacted in 10 mM Tris buffer with dopamine for 4 h at pH 8.5 to establish 8–10 nm thick PD layer over AuNCs (PD@AuNCs), as shown in Fig. 4. The LSPR band of PD@AuNCs shows a small red-shift upon increasing concentration of dopamine, which is assigned to the possible charge transfer between AuNCs and PD.^50^ It is well known that dopamine gets transformed to 5,6-dihydroxyindolines, their dione derivatives, and other related molecules such as semiquinone under alkaline conditions in the presence of well-known polymerization initiators *i.e.* Tris.^51^ Such products remain closely packed to form PD layer on AuNCs (Fig. 4) through strong supramolecular forces like π–π stacking, hydrogen bonding, and charge transfer.^52–54^

Further titration of PD@AuNCs with higher amount of HAuCl_4_ (1 mM) in the presence of reducing agent trimethylamine (TEA) not only leads to formation of AuNPs, but also gradually scratches the inner silver forming AgNPs. As produced Au/AgNPs are embedded in the peeled polymer layer producing nanopeeled AuNCs (NP-AuNCs) as shown in Fig. 4e–h. The formation mechanism of Au/AgNPs involves the stepwise reduction of Au^3+^ to Au^0^ through TEA and PD as reductant and stabilizers.^55^ Meanwhile, the oxidative product of PD in alkaline buffer solution *i.e.* semiquinone reduces the Au^3+^ ions to form AuNPs that deposit on the surface of the preformed seeds aided by TEA.^56^ Subsequently, the oxidation and polymerization of PD forms a capping layer on Au/AgNPs giving rise to nanopeeled assembly, while retaining the AuNCs skeleton/basic frame as shown in Fig. 4e–h. In particular, LSPR observation (Fig. S5a†) revealed that densely packed NP-AuNCs assembly generated strong optical signals having *λ*_max_ at around 490 nm corresponding to combined effects of both Au and AgNPs on plasmonic coupling behaviour as observed previously.^51^

By comparing the FTIR spectra (Fig. S5b†) of PD@AuNCs and NP-AuNCs considerable decrease along with slight shift in intensity of stretching vibrations of O–H and N–H at 3358 cm^−1^ was observed in NP-AuNCs because the –OH of semiquinone is now involved in stabilizing Au/AgNPs.^57^ Meanwhile the decrease in intensity of C–N bending and C–O stretching vibrations of PD in case of NP-AuNCs as compared to PD@AuNCs is also observed at 1595 and 1389 cm^−1^ respectively due to adsorption on metallic surface. These peaks indicate the interaction and stabilization of Au/AgNPs with semiquinone.^11^ Stability of the nanopeeled structure is well correlated by retaining the stretching vibration of C–H, N–H and C=C at 2851 cm^−1^, 1610 cm^−1^, and 1505 cm^−1^, respectively in the benzene ring of the polydopamine.^58^

Hollow AuNCs with porous, thin walls enhance the catalytic activity as they have much larger reactive surface areas allowing both reactants and products to transfer through the surface.^29^ Owing to lower *E*_a_ and relatively higher density introduced by enhancing number of effective catalytic sites in terms of Au/AgNPs, NP-AuNCs would greatly contribute to the stability and catalytic reactivity of nanopeeled assembly. The lower reduction potential of solid AuNPs as compared to AuNCs indicates that the less connected nanoparticles on the surface of the AuNCs are more easily oxidized.^29,46^

**Fig. 5:**
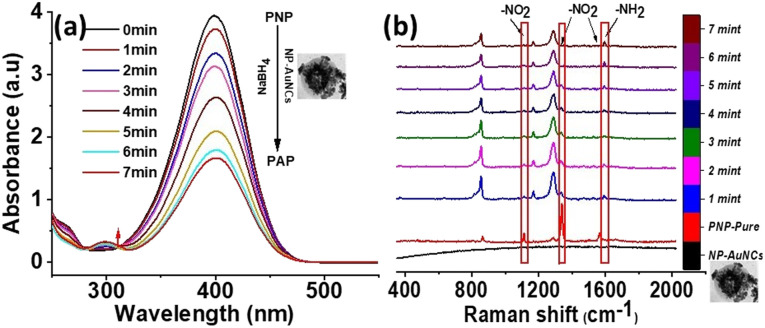
(a) UV-Vis and (b) SERS spectra recorded at various times (0–7 min) during the reduction of PNP to PAP catalysed by NP-AuNCs, prepared by titrating (10 mg) PD@AuNCs with 200 μL (1 mM) HAuCl_4_ in the presence of NaBH_4_. Disappearance of –NO_2_ bands of PNP is accompanied by appearance of –NH_2_ bands of PAP.

Therefore, taking advantage of this property of NP-AuNCs carrying Au/AgNPs embedded in its corona assembly, we reveal precisely enhanced catalytic reduction of PNP. For this purpose, as a model reaction we used NaBH_4_ mediated catalytic hydrogenation of PNP.^59^ Characteristic absorption peak at ∼400 nm due to the absorption of *p*-nitrophenolate ions^29^ was observed after mixing PNP with NaBH_4_ (Fig. 5), which upon addition of NP-AuNCs gradually decreased as the catalytic hydrogenation reaction progressed. Meanwhile, emergence of absorption band at ∼295 nm gradually became more obvious, indicating the formation of *p*-aminophenol (PAP).

We also investigated the catalytic performance of peeled nanoassembly obtained by titrating PD@AuNCs against different volumes of 1 mM HAuCl_4_. Fig. S6† shows the absorbance profiles at 400 nm as a function of time for four volumes of HAuCl_4_: 100, 150, 200 and 250 μL. Data confirmed that volumes of 200 μL and above of HAuCl_4_ could catalyze the reduction of PNP within half time as compared to lower volumes. This is possibly due to (1) maximum thinness of walls of AuNCs by wiping away silver from interiors of half titrated AuNCs, leading to substantially increased catalytic activity; (2) optimum number of Au/AgNPs in the peeled nanoassembly giving rise to less connected nanoparticles on the surface of the AuNCs offering higher reduction capability.^29,46^ However, no or negligible catalytic activity was observed with pure or PD@AuNCs (Fig. S7†).

Reduction of PNP (0.1 mM) to PAP in the presence of NaBH_4_ was also monitored using Raman spectrophotometer at 532 nm. The reaction was completed within the mixing period and peak at 400 nm (nitrophenolate ions) suddenly shifted to *λ*_max_ ∼ 300 nm (PAP). We also used *in situ* SERS to monitor the reduction of PNP in the presence of NP-AuNCs as a catalyst (Fig. 5B). Raman spectrum of PNP exhibits three characteristic vibrational bands at 1108 cm^−1^ (O–N–O stretching), 1336 cm^−1^ (O–N–O stretching), and at 1572 cm^−1^ (phenol-ring). As the catalytic reaction proceeded the intensities of these bands decreased within 7 min with the concomitant emergence of new band at 1591 cm^−1^ that is attributed to PAP. The proposed strategy well integrates the optimal SERS and catalytic properties for highly sensitive monitoring of toxic nitrophenols.

## Conclusions

In short presented synthesis adds benefit over existing protocols to synthesize Ag nanocubes. Comparatively stable S^2−^ producing species Na_2_S_2_O_3_ and soft etchant Cl^−^ emerging from KCl facilitated fabrication of Ag nanocubes within optimum duration of 45 minutes in argon free environment. Principally, effect of both protonic and anionic behaviour of different potassium halides are justified to achieve nanocubes with perfect edges and truncated corners. During transformation of Ag nanocubes to AuNCs modification at an interface of involved metals resulted greatly enhanced SERS signals lead to detect Raman reporter (4MBA) down to pico-molars. The enhanced SERS active probes (Ag nanocubes@4-MBA@AuNCs) served like super sensitive imaging probe for breast cancer cells (MCF-7). Subsequently, MTT assays confirmed the applicable and bio-safety limits of programmed nanoassemblies. Furthermore, the proposed strategy integrates the SERS and catalytic properties with higher sensitivity towards toxic nitrophenols by incorporation of Au/Ag alloy NPs upon triggered oxidative nanopeeling of half-titrated dopamine coated AuNCs. Nevertheless, the developed Ag@4MBA@AuNCs and NP-AuNCs are definite candidates for future studies allied with tumor imaging and control as well as catalytic and analytical performances.

## Author contributions

Farukh Mansoor is the lead author and is involved in basic concept, data curation, funding acquisition, formal analysis, investigation, writing of original draft. Miss Madiha Saeed is mainly involved in MTT assay and other biological handlings. Corresponding authors Shamsa Kanwal and Huangxian Ju guided and lead author during every stage of the implementation plan.

## Conflicts of interest

There are no conflicts to declare.

## Supplementary Material

RA-013-D3RA06344E-s001
