# Supplementary material for: Facile synthesis of gold nanocages with silver nanocubes templates dual metal effects enabled SERS imaging and catalytic reduction
Source: RSC Adv. 2023 Oct 27;13(45):31366–74. doi: 10.1039/d3ra06344e (PMC10603383; doi:10.1039/d3ra06344e)
Supplement: RA-013-D3RA06344E-s001 [file RA-013-D3RA06344E-s001.pdf]

Electronic Supplementary Material (ESI) for RSC Advances.
This journal is © The Royal Society of Chemistry 2023

Supporting Information

Facile Synthesis of Gold Nanocages with Silver Nanocubes Templates Dual Metal Effects Enabled SERS Imaging and Catalytic Reduction

Farukh Mansoor[a,b], Huangxian Ju[c], Madiha Saeed[a,d], Shamsa Kanwal[b*]

[a] Key Laboratory of Magnetic Materials and Devices & Division of Functional Materials and Nanodevices, Ningbo Institute of Materials Technology and Engineering, Chinese Academy of Sciences, Ningbo 315201, P. R. China

[b] Department of Chemistry, Khwaja Fareed University of Engineering and Information Technology, Abu Dhabi Road Rahim Yar Khan, Pakistan

[c] State Key Laboratory of Analytical Chemistry for Life Science, School of Chemistry and Chemical Engineering, Nanjing University, Nanjing 210023, China.

[d] Interdisciplinary Research Centre in Biomedical Materials, COMSATS University, Islamabad, Lahore Campus, Lahore, Pakistan

Email addresses: shamsa.sham@gmail.com

Reagents and Materials. Silver triflouroacetate (CF_3COOAg), potassium iodide (KI), potassium bromide (KBr), potassium chloride (KCl), tetrachloroauric(III) acid ($HAuCl_4 \cdot 3H_2O$), poly(vinylpyrrolidone) (PVP), sodium borohydride ($NaBH_4$), para nitrophenol (PNP), hydrochloric acid (HCl) and triethyl amine (TEA) were purchased from Sinopharm Chemical Reagent Co. Ltd. (Shanghai, China). Dopamine (DP), 4-mercaptobenzoic acid (4MBA), 5-diphenyl-2-H-tetrazolium bromide (MTT), Dulbecco's modified Eagle's medium (DMEM), and

fetal bovine serum (FBS, Gibco) were purchased from Keygen Biotech Co. Ltd. (Nanjing, China). All of the chemicals were used as received without further purification.

Instrumentation. Transmission electron microscopic (TEM) images and energy dispersive X-ray spectra (EDS) were recorded on Tecnai F20 transmission electron microscope operated at 200 kV. Surface morphology and element distribution of Ag nanocubes and AuNCs were recorded on field emission scanning electron microscope (FESEM, S-4800). FTIR spectra of samples were recorded on Nicolet Is10 (USA) spectrometer. For powder X-ray diffraction (XRD) of the samples Rigaku Rotaflex Dmax2200 diffractometer (Japan) with Cu Kα radiation (λ = 1.54056 Å) was used. The elemental composition and chemical valence states of Ag nanocubes and AuNCs were analyzed by X-ray photoelectron spectroscopy (XPS) recorded on Kratos Axis Ultra DLD with a nominal energy resolution of 0.48 eV at room temperature. Absorption spectra were recorded on UV-3600 UV-Vis-NIR spectrophotometer made by Shimadzu, Japan. Raman and SERS spectra were obtained with Renishaw in Via Reflex instrument, England. Lasers 633 and 532 nm lines from an Ar-Kr ion laser, and laser 785 nm line from semiconductor laser were used as excitation sources. The cell viabilities in the MTT assay were measured through a BioTek ELX 800 microplate reader.

Calculation of Enhancement Factor. We used the SERS peak at 1077 cm^{-1} of 4MBA reporter molecule to calculate the SERS enhancement factor (EF) using the following equation:

$$EF = (I_{SERS} \times N_{bulk})/I_{bulk} \times N_{SERS} \qquad (1)$$

I_{SERS} and I_{bulk} are the intensities of the same band for the SERS and ordinary spectra from a bulk sample, N_{bulk} and N_{SERS} are the numbers of bulk molecules probed for a bulk sample and those molecules probed in SERS, respectively.

Note that I_{SERS} and I_{bulk} were determined by the area of 1077 cm^{-1} bands while N_{bulk} was determined from the ordinary Raman spectrum of a 0.01mM 4MBA solution and the focal volume of our Raman system. Specifically, we evaluated the EF by using 4MBA@AuNCs and Ag@4MBA@AuNCs. Based on the SERS spectra shown in Figure 3, we measured the I_{SERS}. For determining the number of molecules probed in SERS, we assumed that the surface was covered by a complete monolayer of 4MBA molecules. Hence, theoretically the calculated value provides a maximum number of molecules and thus calculated EF values necessarily represent an underestimate of the actual value.

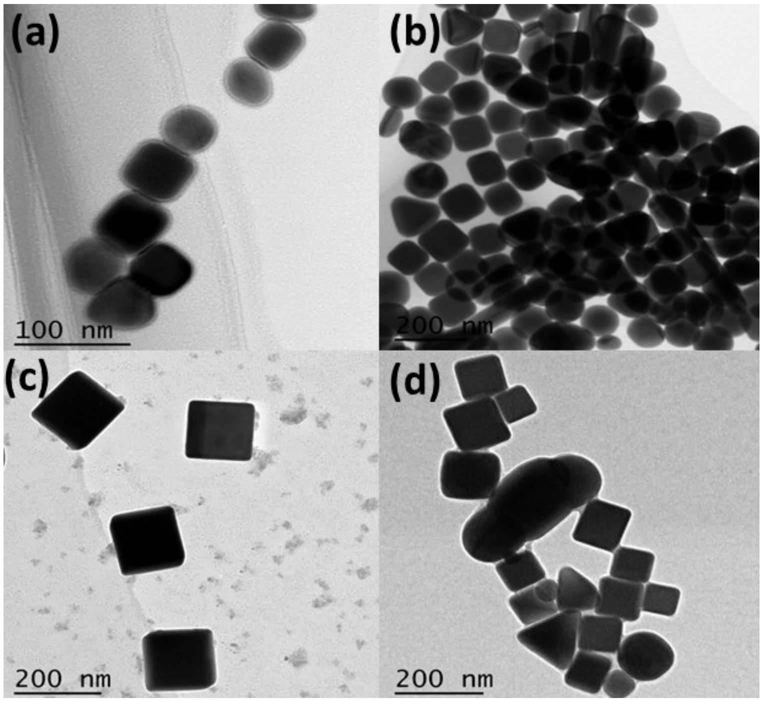

Figure S1. TEM images of Ag nanocubes with a) 40, b) 50, c) 55 and d) 60 R of 3 mM KCl.

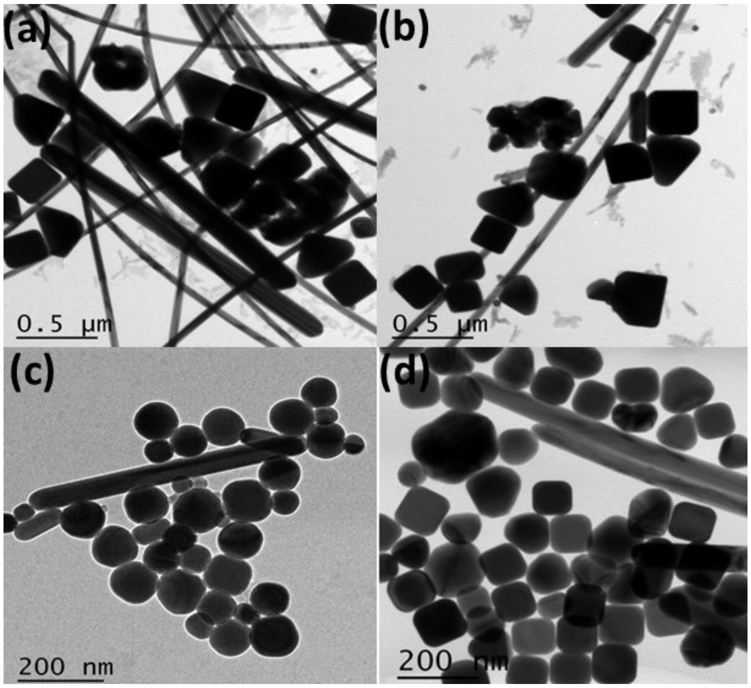

Figure S2. TEM images of as synthesized Ag nanocubes with a) 50, b) 60 µL KBr and c) 50, d) 60 µL KI.

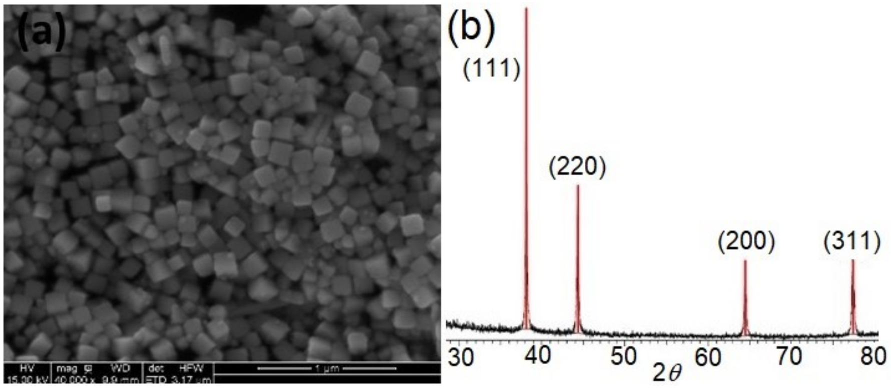

Figure S3. SEM image and XRD spectrum of as synthesized Ag nanocubes.

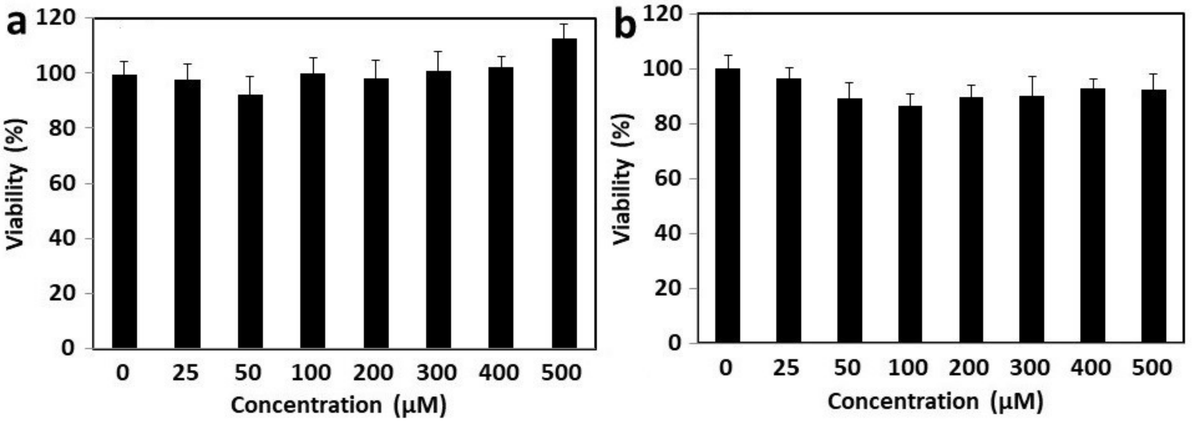

Figure S4. Viabilities of MCF-7 cells after incubated with a) 4MBA@AuNCs and b) Ag@4MBA@AuNCs at various concentrations for 24 h recorded at 550 nm.

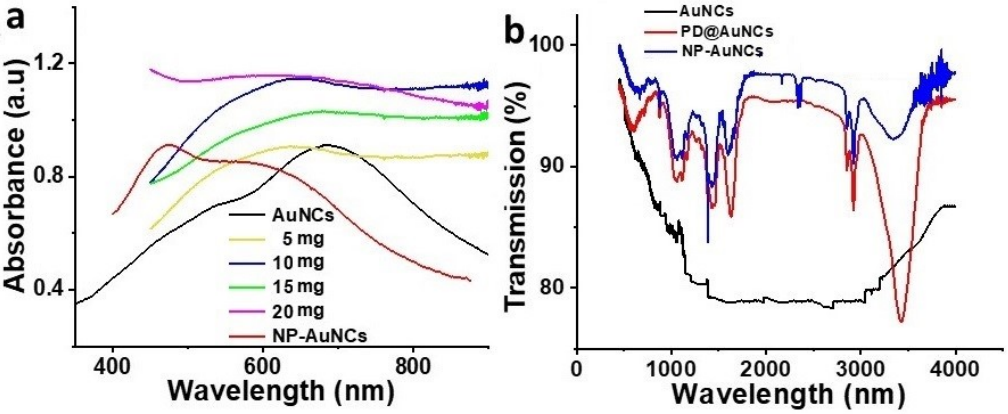

Figure S5. a) UV-vis and b) FTIR spectra of AuNCs, PD@AuNCs and NP-AuNCs. PD@AuNCs: 5-20 mg PD coated AuNCs. NP-AuNCs: PD@AuNCs upon re-titration with 200 μL $HAuCl_4$.

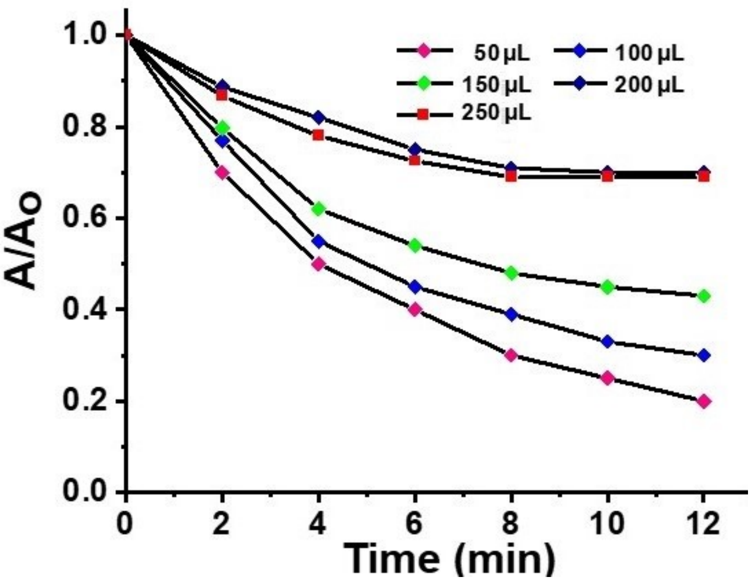

Figure S6. Absorption (normalized against the initial point) of NP-AuNCs at 400 nm as a function of reaction time. NP-AuNCs: PD@AuNCs upon re-titration with 50-250 µL 1 mM $HAuCl_4$.

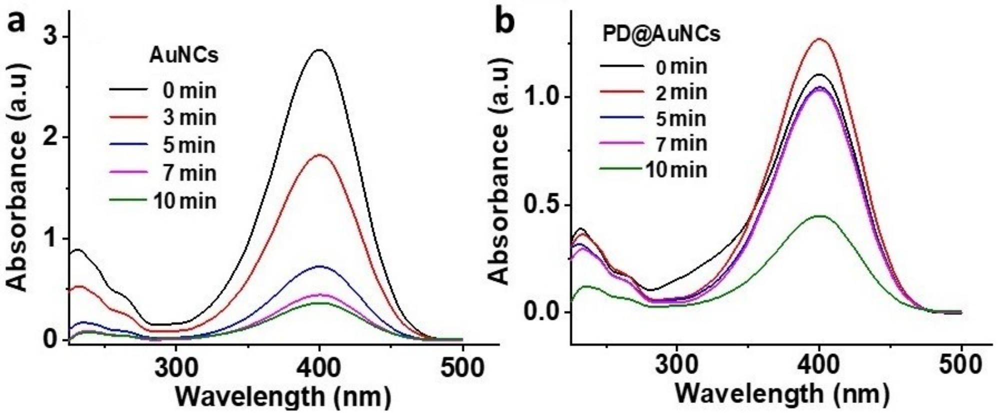

Figure S7. UV-vis spectra of 500 µl PNP (1 µM) at different catalytic reaction times in the presence of 50 µL a) AuNCs and b) PD@AuNCs (10 mg PD coated AuNCs).

Table S1: Effect of KCl concentration on LSPR of Ag nanocubes.

**$Na_2S_2O_3$** (3 mM, 1.2 mL)	**KCl** (3 mM)	**LSPR (λ_{max}/Abs)**
	40 µL	459/2.01
	50 µL	463/1.24
	55 µL	490/3.80
	60 µL	510/1.67

Table S2: Optimization of $Na_2S_2O_3$ and salt concentration for synthesis of Ag nanocubes.

**KX**	**$Na_2S_2O_3$ (3 mM, mL)**	**KX (3 mM, µL)**	**LSPR (λmax/Abs)**
KCl (3 mM)	1	50	455/0.78
	1.2	50	463/1.24
	1.4	55	478/1.77
KBr (3 mM)	1.2	50	544/0.9
	1.2	100	491/2.91
KI (3 mM)	1	50	548/0.14
	1	60	542/0.29
	1.2	50	595/0.21
	1.2	60	559/0.15
